# *AetMYC1*, the Candidate Gene Controlling the Red Coleoptile Trait in *Aegilops tauschii* Coss. Accession As77

**DOI:** 10.3390/molecules22122259

**Published:** 2017-12-18

**Authors:** Dong Cao, Guangji Ye, Yuan Zong, Bo Zhang, Wenjie Chen, Baolong Liu, Huaigang Zhang

**Affiliations:** 1 Qinghai Provincial Key Laboratory of Crop Molecular Breeding, Xining 810008, China; caod.08@163.com (D.C.); zhangbo@nwipb.cas.cn (B.Z.); cwj60905@163.com (W.C.); 2 State Key Laboratory of Plateau Ecology and Agriculture, Qinghai University, Qinghai, Xining 800010, China; 13997089595@163.com (G.Y.); laughing1898@icloud.com (Y.Z.); 3 Northwest Institute of Plateau Biology, University of Chinese Academy of Sciences, Beijing 100049, China; 4 Key Laboratory of Adaptation and Evolution of Plateau Biota, Northwest Institute of Plateau Biology, Chinese Academy of Sciences, Xining 810008, China

**Keywords:** *Ae. tauschii*, coleoptile, anthocyanin biosynthesis, *bHLH* transcription factor, *MYB* transcription factor

## Abstract

The red coleoptile trait can help monocotyledonous plants withstand stresses, and key genes responsible for the trait have been isolated from *Triticum aestivum*, *Triticum urartu*, and *Triticum monococcum*, but no corresponding research has been reported for *Aegilops tauschii*. In this research, transcriptome analysis was performed to isolate the candidate gene controlling the white coleoptile trait in *Ae. tauschii*. There were 5348 upregulated, differentially-expressed genes (DEGs) and 4761 downregulated DEGs in red coleoptile vs. white coleoptile plants. Among these DEGs, 12 structural genes and two transcription factors involved in anthocyanin biosynthesis were identified. The majority of structural genes showed lower transcript abundance in the white coleoptile of accession ‘As77’ than in the red coleoptile of accession ‘As60’, which implied that transcription factors related to anthocyanin biosynthesis could be the candidate genes. The *MYB* and *MYC* transcription factors *AetMYB7D* and *AetMYC1* were both isolated from *Ae. tauschii* accessions ‘As60’ and ‘As77’, and their transcript levels analyzed. The coding sequence and transcript level of *AetMYB7D* showed no difference between ‘As60’ and ‘As77’. *AetMYC1p* encoded a 567-amino acid polypeptide in ‘As60’ containing the entire characteristic domains, bHLH-MYC_N, HLH, and ACT-like, belonging to the gene family involved in regulating anthocyanin biosynthesis. *AetMYC1w* encoded a 436-amino acid polypeptide in ‘As77’ without the ACT-like domain because a single nucleotide mutation at 1310 bp caused premature termination. Transient expression of *AetMYC1p* induced anthocyanin biosynthesis in ‘As77’ with the co-expression of *AetMYB7D*, while *AetMYC1w* could not cause induced anthocyanin biosynthesis under the same circumstances. Moreover, the transcript abundance of *AetMYC1w* was lower than that of *AetMYC1p*. *AetMYC1* appears to be the candidate gene controlling the white coleoptile trait in *Ae. tauschii*, which can be used for potential biotech applications, such as producing new synthetic hexaploid wheat lines with different coleoptile colors.

## 1. Introduction

The coleoptile is the pointed protective sheath covering the emerging shoot in monocotyledonous plants. The exact function of the red coleoptile during the life cycle of plants is unclear. Preliminarily results from previous studies have demonstrated its function is to protect the emerging shoot from strong light, drought, and cold [1,2]. Unlike the true leaf rolled up within, the pre-emergent coleoptile does not accumulate significant proto-chlorophyll or carotenoids, and so it is generally either very pale (“white”) or red. The majority of common wheat (*Triticum aestivum*) cultivars have white coleoptiles, while wild wheat relatives usually carry red coleoptiles [3,4,5]. Since it is an easy trait to observe, the coleoptile color trait has been used as a character describing wheat varieties. Eight anthocyanins have been identified in the red wheat coleoptile, which indicates that the presence of red pigmentation is connected with the synthesis of anthocyanins in the wheat coleoptile.

The main structural genes involved in anthocyanin biosynthesis include phenylalanine ammonia-lyase (*PAL*), cinnamate-4-hydroxylase (*C4H*), 4-coumarate: CoA ligase (*4CL*), chalcone synthase (*CHS*), chalcone isomerase (*CHI*), flavanone 3-hydroxylase (*F3H*), flavonoid-3’-hydroxylase (*F3’H*), flavonoid-3’,5’-hydroxylase (*F3′5′H*), flavonol synthase (*FLS*), dihydrofavonol-4-reductase (*DFR*), leucoanthocyanidin dioxygenase (*LDOX*), and flavonoid-3-*O*-glucosyltransferase (*UFGT*) [6,7]. These structural genes are regulated mainly by two major classes of transcription factors, the *R*/*B* family (basic helix–loop–helix (*bHLH*) type) and the *C1*/*Pl* family (*MYB* type). bHLH (MYC) proteins contained the three important domains, including bHLH-MYC_N, HLH, and ACT-like, while MYB protein includes R2-MYB, R3-MYB, and transcript activator domains. MYC and MYB proteins can form the complex for exercising their transcriptional functions [8,9]. The bHLH-MYC_N domain is required for the protein-protein interactions with *MYB* transcription factors, the HLH domain facilitates DNA binding, and the ACTlike domain interacts with the RNA polymerase II machinery and then initiates transcription [8,9]. All these genes (structural or regulatory) are important for the anthocyanin biosynthesis pathway. The inactivation of any one gene could block the entire metabolic pathway, and cause the pale phenotype in plant tissue. Allelic variants of the *MYB* and *bHLH* genes are more common causes of color variation in plants than are variants of the anthocyanin structural genes [10].

Anthocyanin pigmentation of common wheat coleoptiles is controlled by three genes (*Rc1*, *Rc2*, *Rc3*) on the short arm of homeologous chromosomes 7A, 7B, and 7D, respectively [4]. Wheat cultivars carrying any one of these three genes exhibit the red coleoptile trait, whereas cultivars with the *rc1rc2rc3* genotype show no pigmentation (white coleoptile trait) [11]. Recently, *Rc1*, *Rc2*, and *Rc3* were found to encode three *MYB* transcription factors *TaMYB-A1*, *TaMYB-B1*, and *TaMYB-D1* in *Triticum aestivum* [12,13,14]. The loss-of-function mutation in the gene homologous to *Rc* in *T. urartu* and *T. aestivum* also resulted in the white coleoptile trait [5,14]. The white coleoptile trait also exists in *Ae. tauschii*, but the gene responsible for the trait has not been identified.

High-throughput RNA sequencing (RNA-Seq) provides an approach for detecting novel transcripts, single nucleotide polymorphisms, small RNAs and alternate splicing products, as well as sense and antisense transcripts [15,16]. This has the advantage of measuring gene expression levels, and obtaining the corresponding nucleotide sequences without reference sequences. In this paper, RNA-Seq was employed to compare the expression of the structural genes and transcription factors involved in anthocyanin biosynthesis in red coleoptiles of ‘As60’ and white coleoptiles of ‘As77’. Based on transcriptome information, the *bHLH* and *MYB* transcription factors, *AetMYC1* and *AetMYB7D,* were isolated, in order to identify the key gene responsible for the white coleoptile trait in ‘As77’.

## 2. Results

### 2.1. Transcriptome Analyses of Red and White Coleoptiles

After filtering, 60.85 Mb reads from the red coleoptiles of ‘As60’ and 38.85 Mb from the white coleoptiles of ‘As77’ remained, with Q30 percentages of 90.01% and 89.82%, respectively. The high-quality reads were aligned to assemble 83,385 unigenes with an average length of 987 nt and an N50 length of 1644 nt, using Trinity software (2.2.0, GitHub, Inc, San Francisco, CA, USA). Unigenes putatively differentially expressed between red and white coleoptiles were identified on the basis of fragments per kb per million reads (FPKM) values, calculated from the read counts mapped onto the reference transcriptome. A total of 10,109 unigenes were differentially expressed between red and white coleoptiles, according to a comparison of expression levels with FDR ≤ 0.001 and |log_2_Ratio| ≥ 1 (Figure S1A). Using the red coleoptile as the reference, 5348 up-regulated unigenes (with greater levels of expression in white coleoptiles) and 4761 down-regulated unigenes (with lower levels of expression in white coleoptiles) were identified.

**Figure 1:**
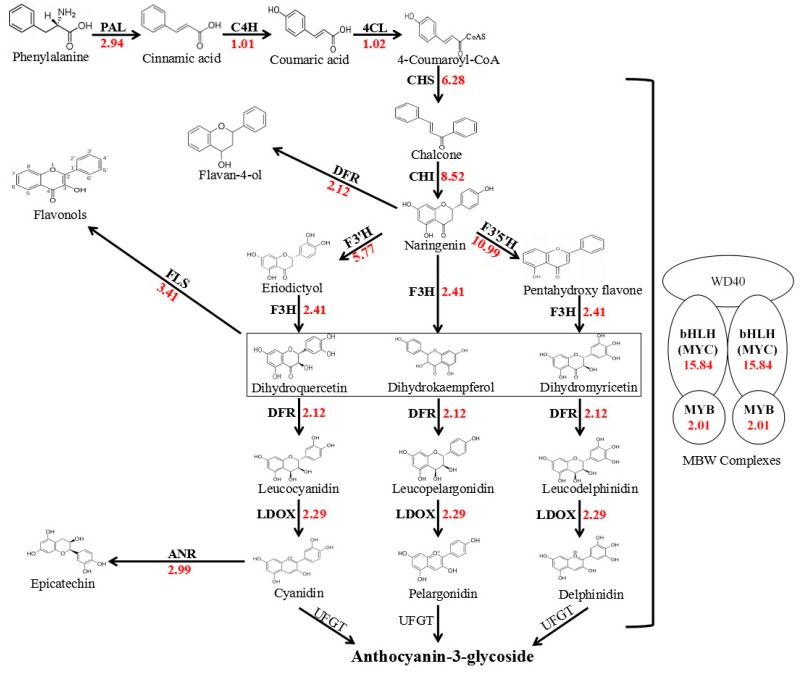
The expression differences of structural genes involved in anthocyanin biosynthesis. The arrow shows the metabolic stream. Abbreviations of left or upward arrows represent the genes catalyzing the progress. The light abbreviations represent that these genes were not found among the assembly unigenes. The red number represents the expression in the red coleoptile relative to that in the white coleoptile (expression (red)/expression (white)).

To further clarify the key genes responsible for the white coleoptile trait, 12 structural genes and two transcription factors (Figure 1, Table S1), related to anthocyanin biosynthesis were selected for a BLAST search of the assembled unigene database. *PAL*, *C4H*, *4CL*, *CHS*, *CHI*, *F3H*, *F3′H*, *F3′5′H*, *DFR*, *LDOX*, *ANR*, *MYB*, and *MYC* could be detected in transcriptome analysis (Figure 1), only *UFGT* being absent. In addition, *DFR*, *F3′H*, *F3′5′H*, and *FLS* competed for the same substrate to yield other compounds. Upstream (*PAL*) and midstream (*CHS*, *CHI*, *DFR*, *LDOX*) genes had higher expression levels, compared to downstream genes (Table S1). All of the structural genes exhibited higher expression levels in the red coleoptiles, from 1.01 to 10.99 times higher than that in the white coleoptiles, which should imply that the transcription factors included the key gene for the white coleoptile trait. The transcript levels of *MYB* in red coleoptiles were similar to those in white coleoptiles (Table S1). The transcript levels of the *bHLH* transcription factors in red coleoptiles were 15.84 times greater than in white coleoptiles. The greatest FPKM value was 16.43 in red coleoptiles, while the greatest value was 0.82 in white coleoptiles (Table S1). Moreover, several single-nucleotide polymorphism (SNP) differences existed between the coding sequences of *AetMYC1* from the two accessions.

### 2.2. Molecular Characteristics of AetMYB7D and AetMYC1

Based on the assembly sequences obtained from transcriptome analysis, *AetMYC1* and *AetMYB7D* were isolated from ‘As60’ and ‘As77’ for further evaluating their role in determining the white coleoptile trait of ‘As77’. *AetMYB7D* had the same nucleotide sequences in ‘As77’ (Genebank accession: MG495090) and ‘As60’ (Genebank accession: MG495089), and encoded the same protein as *TaMYB7D* (Genebank accession: KP136432), which is located on chromosome 7D of common wheat.

**Figure 2:**
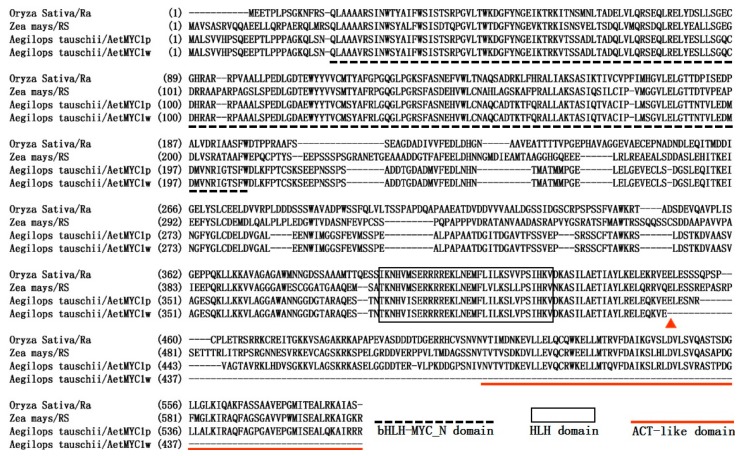
Amino acid sequence alignment of bHLH transcription factors AetMYC1, RS and Ra. The black dotted lines represent the conserved bHLH-MYC_N domain, the black rectangle represents the HLH domain, and the red dotted lines represent the ACT-like domain. The red triangle represents the location of the stop codon.

**Figure 3:**
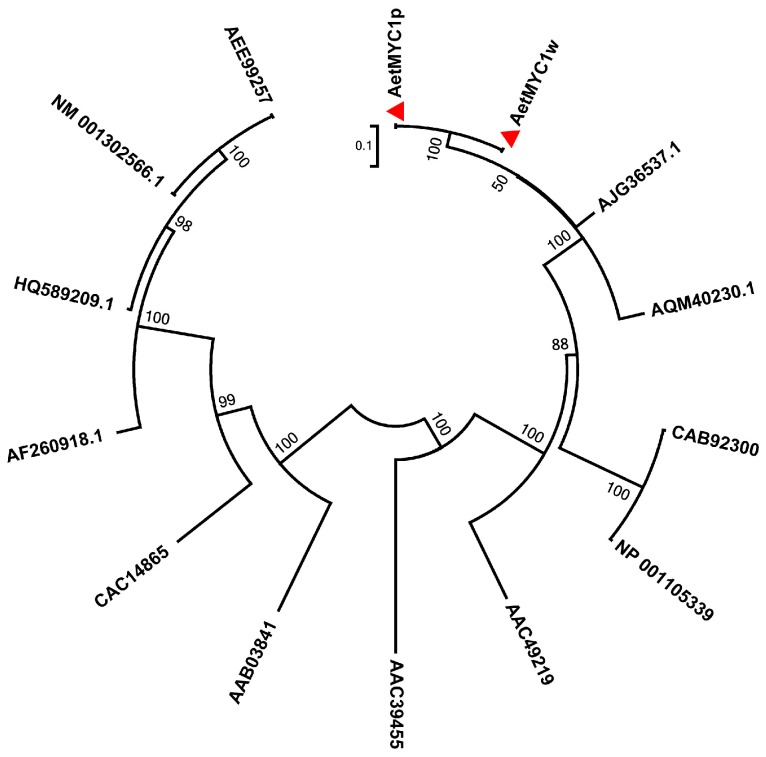
Phylogenetic relationships between AetMYC1 and bHLHs in other species. The accession numbers of these proteins are as follows: AJG36537.1: *Triticum aestivum*/TaMYC1; AQM40230: *Hordeum vulgare*/HvMYC1; AAC49219: *Oryza sativa*/Ra; CAB92300: *Zea mays*/Hopi; NP_001105339: *Zea mays*/SN; AAB03841: *Zea mays*/IN1; CAC14865: *Arabidopsis thaliana*/TT8; AEE99257: *Nicotiana tabacum*/AN1a; NM_001302566.1: *Nicotiana tabacum*/AN1-like; HQ589209.1: *Nicotiana tabacum*/AN1b; AF260918.1: *Petunia x hybrida*/AN1; AAC39455: *Petunia x hybrida*/JAF13.

The coding sequence of *AetMYC1p* (Genebank accession: MG495087) from ‘As60’ was 1704 bp long, and the 567 amino acid primary structure of the protein encoded contained the integrated bHLH-MYC_N domain of 178 amino acids, which is essential for protein-protein interaction, the HLH domain of 32 amino acids, which could facilitate protein-DNA binding, and the ACT-like domain of 78 amino acids, which initiates transcription (Figure 2). The phylogenetic tree was constructed by the neighbor-joining method, using the amino acid sequences of *bHLH* transcription factors (Figure 3). bHLH proteins from the same species were clustered together. AetMYC1 was assigned to the same branch as AJG36537.1 (TaMYC1), AQM40230 (HvMYC1), AAC49219 (Ra), CAB92300 (Hopi), and NP_001105339 (SN) (Figure 3). These proteins regulate anthocyanin biosynthesis in *T. aestivum* [17], *Hordeum vulgare* [18], *Oryza sativa* [19], and *Zea mays* [20], which suggests that *AetMYC1* should carry out the function of regulating anthocyanin biosynthesis in *Ae. tauschii*. The location of *AetMYC1* was on chromosome 2D, based on the BLASTX algorithm with an *E*-value < 1 × 10^−5^ and the reference database (*Triticum aestivum*. IWGSC1 proseq. 3.1.). *AetMYC1* should be the homologous to *TaMYC1* [17], one of the complement of genes controlling red pericarp trait in common wheat. In the *Ae. tauschii* genome database, *AetMYC1* shows a high sequence identity to *AetMYC1.4* and *AetMYC1.5* [21] (Table S2). The sequence of *AetMYC1w* (Genebank accession: MG495088) from ‘AS77’ had three nucleotide differences from that of *AetMYC1p* from ‘As60’ (Table S3), and encoded 436 amino acids (compared to 567 amino acids encoded by *AetMYC1p*) because a single nucleotide mutation at 1310 bp caused premature termination of translation of the encoded protein (Figure 2). As a result of the premature termination, AetMYC1w had lost the ACT-like domain (Figure 2).

### 2.3. Differences of Transcript Abundance and Regulatory Activity of AetMYB7D and AetMYC1

**Figure 4:**
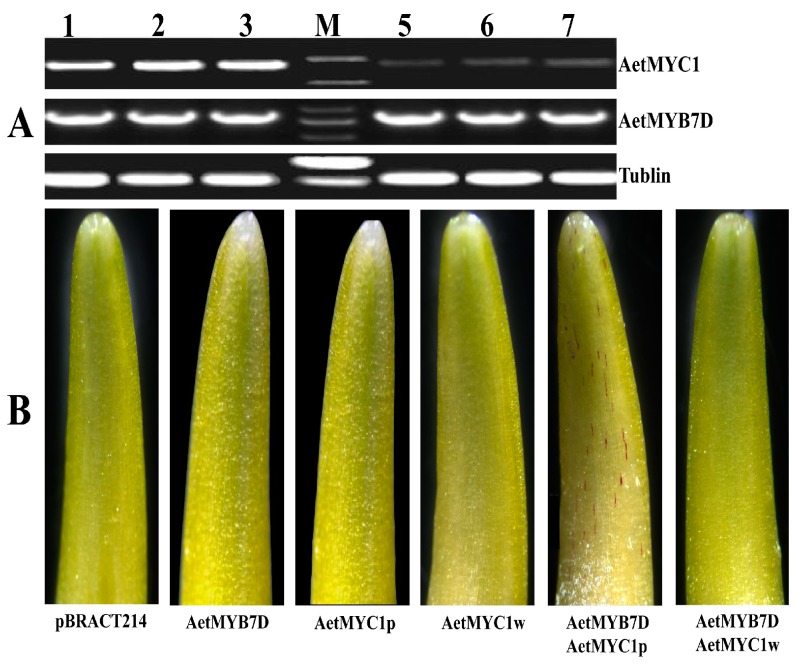
Functional verification of *AetMYC1* and *AetMYB7D*. (**A**) Expression profiles of *AetMYC1* and *AetMYBD* in red and white coleoptiles. Lanes 1–3 were from the red coleoptile allele *AetMYC1p*, lanes 5–7 were from the white coleoptile allele *AetMYC1w*; (**B**) The coleoptile two days after particle bombardment. AetMYB7D, AetMYC1p, and AetMYC1w represent transformations with the plasmids pBRACT214-AetMYB7D, pBRACT214-AetMYC1p, and pBRACT214-AetMYC1w, respectively.

Transcriptome analysis revealed a difference in transcript abundance between *AetMYB7D* and *AetMYC1*, with RT-PCR being employed to verify this transcript difference. The transcript abundance of *AetMYC1* in white coleoptiles was markedly lower than that in red coleoptiles, while the transcript abundance of *AetMYB7D* in white coleoptiles was similar to that in red coleoptiles (Figure 4A). This result was close to that obtained from transcriptome analysis.

The transcription factors *bHLH* and *MYB* were co-expressed, and could function together to induce anthocyanin biosynthesis in wheat coleoptiles [22]. *AetMYB7D* had the same base sequences in ‘As60’ and ‘As77’, and encoded the same protein as *TaMYB7D*, with the function of regulating anthocyanin biosynthesis. One transient expression construct, pBRACT214-AetMYB7D, was shown to express a functional *MYB* transcription factor in a transient expression experiment to identify any function differences between *AetMYC1p* and *AetMYC1w*. The transient expression of *AetMYC1r* induced anthocyanin biosynthesis in the white coleoptile cells of *T. aestivum* ‘Opata’ in the presence of *AeMYB7D*, while *AeMYC1w* did not induce anthocyanin biosynthesis in the presence of *AetMYB7D* in the same system (Figure 4B). The red cell numbers were counted in the coleoptile bombarded (Table S4) Only *pBRACT214*, *AetMYC1p*, *AetMYC1w*, and *AetMYB7D* yielded no red cells in the coleoptile (Figure 4B). This meant that *AetMYC1p* worked by regulating anthocyanin biosynthesis, while *AeMYC1w* did not.

## 3. Discussion

Our previous research had found that all of the *T. urartu* and *T. monococcum* cultivars had red coleoptiles, except one *T. urartu* accession [5], while most cultivars of common wheat had white coleoptiles. The results were similar to previous results. All *Ae. tauschii* cultivars collected from Pakistan, Afghanistan and Iran were found to carry red coleoptiles [3], and approximately 71% common wheat cultivars carried white coleoptiles (https://wheat.pw.usda.gov/ggpages/awn) [4]. These results suggested that red coleoptiles can help *T. urartu*, *T. monococcum*, and *Ae. tauschii* cope with adversity and are useful for surviving under natural conditions. Under artificial conditions, such as modern agriculture, stresses occur less frequently because the farmer controls the sowing date, and provides enough resources for germination, such as water and fertilizer. The red coleoptile may not be so necessary for plant survival for cultivated common wheat. In *Ae.tauschii*, only one accession exhibited white coleoptiles out of 144 accessions tested.

The majority of anthocyanin biosynthesis structural genes showed a lower transcript abundance in the white coleoptiles of ‘As77’ than in the red coleoptiles of ‘As60’. These structural genes are regulated primarily by the MYB-bHLH-WD40 complex [21], and the allelic variants of *MYB* and *bHLH* genes are more commonly responsible for color variation in plants [10]. In this study, the *MYB* transcription factor *AetMYB7D* was shown to regulate anthocyanin biosynthesis in a transient expression experiment, and encoded the same protein as did *TaMYB7D*. *TaMYB7D* encodes a functional *MYB* transcription factor, regulating anthocyanin biosynthesis, and has been proven to be *Rc3*, controlling the red coleoptile trait in common wheat [13]. *AetMYB7D* had the same nucleotide sequence and similar expression level in the coleoptiles of ‘As60’ and ‘As77’. The homeologous genes *TaMYB-A1*, *TaMYB-B1*, and *TaMYB-D1* had been shown to control coleoptile color in common wheat in previous research [12,13,14], but *AetMYB7D* could not be the key gene for the white coleoptile trait in *Ae. tauschii*.

The bHLH transcription factor plays a key role in the anthocyanin biosynthesis pathway. Loss of function of a bHLH transcription factor could produce the pale traits, such as white coleoptiles [8,23,24,25,26,27,28,29]. *AetMYC1*, encoding a *bHLH* transcription factor, has two alleles, *AetMYC1p* and *AetMYC1w*. AetMYC1p had the characteristic function domains (including bHLH-MYC_N, HLH, and ACT-like), and had a strong similarity to RS and Ra [8,25], while AetMYC1w had lost the ACT-like domain. Transient expression studies revealed that AetMYC1p could regulate anthocyanin biosynthesis with the assistance of AetMYB7D, but that AetMYC1w could not. The expression level of *AetMYC1p* was significantly higher than that of *AetMYC1w*. Loss of function or low expression level could impede anthocyanin biosynthesis in the coleoptiles, and cause production of the white coleoptile trait. Taking into consideration all of the above results, *AetMYC1* appears to be the key gene responsible for the white coleoptile trait in ‘As77’. Moreover, the new functional *bHLH* transcript factor *AetMYC1* could also be used for obtaining transgenic plants with high anthocyanin content. Common wheat originated from natural hybridization between *Triticum turgidum* (2*n* = 4*x* = 28, AABB) and *Ae. tauschii* (2*n* = 2*x* = 14, DD). By replicating the origin of common wheat, synthetic allohexaploid wheat lines have been generated, using *T. turgidum* and *Ae. tauschii* [28,29]. The red coleoptile trait was speculated to help wheat withstand some environmental stresses, but should be negative to yield under good cultivation conditions. Since the key gene controlling the white coleoptile trait of *Ae. tauschii* was not homologous to the *Rc* genes, the cross between two white coleoptile parents, *T. turgidum* and *Ae. tauschii,* has a high probability of producing common wheat with the new red coleoptile trait. Our result should provide some guidelines and materials for producing new synthetic hexaploid wheat lines with different coleoptile colors.

## 4. Materials and Methods

### 4.1. Plant Materials and Phenotype Scans

A total of 144 *Ae. tauschii* accessions were obtained from the SDA-ARS National Small Grains Collection [30] and the Triticeae Research Institute, Sichuan Agricultural University, China (Table S5). Ten seeds were germinated for surveying the coleoptile color according to the method described by Ye et al. [7]. ‘As77’ was the only accession with white coleoptile (Table S5; Figure S1B). ‘As60’, an *Ae. tauschii* accession carrying red coleoptiles, was chosen as the control for identifying the key gene for the white coleoptile trait in ‘As77’ (Figure S1B).

### 4.2. Genomic DNA, Total RNA, and cDNA Preparation

Genomic DNA was extracted from coleoptiles of five-day-old seedlings, using the cetyltrimethylammonium bromide method [31]. Total RNA was isolated from coleoptiles of five-day-old seedlings with the TIANGEN RNAprep Pure Plant Kit (Tiangen Company, Beijing, China). Total RNA of each sample was quantified and qualified by Agilent 2100 Bioanalyzer (Agilent Technologies, Palo Alto, CA, USA), NanoDrop (Thermo Fisher Scientific, Wilmington, DE, USA) and 1% agrose gel. cDNA was obtained from total RNA using the Thermo RevertAid First Strand cDNA Synthesis Kit (Thermo Fisher Scientific, St. Louis, MO, USA).

### 4.3. Transcriptome Analysis

The cDNA library preparations from coleoptiles were constructed according to the manufacturer’s protocol for mRNA-Seq sample preparation (Illumina, Inc., San Diego, CA, USA). The cDNA library products were sequenced by Illumina paired-end sequencing technology with read lengths of 150 bp on the Illumina HiSeq X instrument by Genewiz Biotechnology Co., Ltd. (Suzhou, China). The raw sequence reads were stored in the National Center for Biotechnology Information (NCBI) SRA database with the accession number SUB3213723.Before assembly, the raw paired-end reads were filtered to obtain high-quality clean reads. Low-quality sequences were removed, including sequences with ambiguous bases (denoted with more than 5% “N” in the sequence trace) and low-quality reads (the rate of reads in which a quality value ≤10 is greater than 20%) and reads with adaptors. After filtering was completed, the high-quality reads were assembled by Trinity, with default parameters to construct unique consensus sequences based on the sequences from both the red and white coleoptile genotypes [32]. Unigenes that were differentially expressed between red and white coleoptile genotypes were analyzed, using chi-square tests with IDEG6 software (University of Padua, Padua, Italy) [33]. The unigene expression level was calculated using the fragments per kb per million reads (FPKM) values. The false discovery rate (FDR) method was introduced to determine the threshold *p*-value at FDR ≤ 0.001, and the absolute value of |log_2_Ratio| ≥ 1 was used as the threshold to determine the significance of the differential expression of the unigenes.

The genes related to anthocyanin biosynthesis in Kyoto Encyclopedia of Genes and Genomes (KEGG) pathways [34] were collected and aligned to the unigenes from a transcriptome mixture of red and white coleoptiles, using the BLASTX algorithm with an *E*-value of <1 × 10^−5^.

### 4.4. Gene Isolation

PCR amplifications were carried out using high-fidelity Phusion DNA polymerase (Thermo Fisher Scientific, USA) in the GeneAmp PCR System 9700 (Applied Biosystems, Waltham, MA, USA). The PCR products were confirmed by electrophoresis in 1.0% agarose gel, and bands of the expected size were purified from the gel using an EasyPure Quick Gel Extraction Kit (TransGen, Bejing, China). The targeted DNA fragments were ligated into PGEMT Easy Vector (Promega, Madison, WI, USA), which were transformed into *Escherichia coli* DH 5a competent cells. The positive clones were picked out and sent to a commercial company (Huada Gene, Beijing, China) for sequencing. Primers used in the study are listed in Table S5.

### 4.5. Transient Expression

The transient expression vector pBRACT214 for vector construction was supplied by the John Institute Centre, Norwich, UK. The transient expression plasmids (pBRACT214-*AetMYC1p, pBRACT214-AetMYC1w*, *pBRACT214*-*AetMYB7D*) were constructed by putting *AetMYC1p*, *AetMYC1w*, and *AetMYB7D* under the control of the maize ubiquitin promoter with the Gateway Cloning Kit (Thermo Fisher Scientific, Shanghai, China). The plasmids were delivered into the white coleoptiles of the common wheat cultivar Opata by particle bombardment [22]. All of the treated coleoptiles from the transient expression study were observed and photographed using a stereoscope (Leica Co., Wetzlar, Germany).

### 4.6. Expression Profiles

The coleoptiles used for the expression profile study were collected two-day after germination and prepared according to the procedure used by Himi et al. [11]. “Light treatment” meant that the coleoptiles were grown in a light incubator (light intensity 100 µmol/s/m^2^, 23 °C, 16/8 h). RT-PCR (reverse transcription PCR) was performed with primers Tubulin-F and Tubulin-R for the tubulin gene to standardize the amount of cDNA template. Primers used are listed in Table S6.

### 4.7. Bioinformatic Analysis

The assembly and alignments of AetMYC1, AetMYB7D, and bHLH protein sequences were conducted using the Vector NTI 10 software package (Thermo Fisher Scientific, USA). The primers were designed using Primer5 software (Premier Biosoft, Palo Alto, CA, USA). The phylogenetic tree was constructed with MEGA5 software (Tokyo Metropolitan University, Hachioji, Tokyo, Japan), using the neighbor-joining method [35].

## Supplementary Materials

The following are available online. Figure S1. Genes differentially expressed between red and white coleoptile; Table S1. Information on unigenes associated with anthocyanin biosynthesis in coleoptiles; Table S2. Homologs of AetMYC1 found in URGI database in *Ae. Tauschii*; Table S3. The locations of single nucleotide polymorphisms (SNPs) in *AetMYC1* alleles; Table S4. The number of red coleoptile cells after bombardment; Table S5. Information on *Ae. tauschii* accessions used in this study; Table S6. Names and sequences of the primers used in this study.
