# Supplementary material for: AetMYC1, the Candidate Gene Controlling the Red Coleoptile Trait in Aegilops tauschii Coss. Accession As77
Source: Molecules. 2017 Dec 18;22(12):2259. doi: 10.3390/molecules22122259 (PMC6149708; doi:10.3390/molecules22122259)
Supplement: Supplementary file 1 [file molecules-22-02259-s001.pdf]

molecules

MDPI

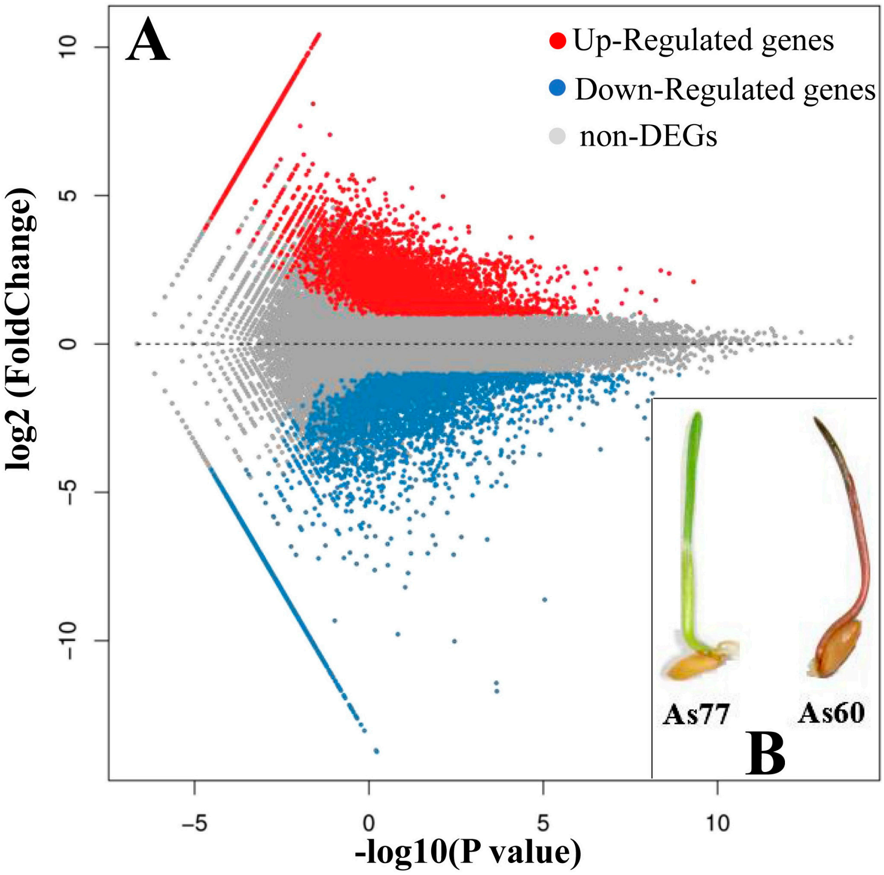

Figure S1. Genes differentially expressed between red and white coleoptile.

Table S1. Information on unigenes associated with anthocyanin biosynthesis in coleoptiles.

Gene	KEGG orthology	KEGG enzyme	Gene ID	Length	Red-Expression	White-Expression	log_2FoldChange (white/red)	FDR	Up/Down-Regulation (white/red)
PAL	K10775	EC:4.3.1.24	*CL3234.Contig4_All*	753	13.55	3.37	2.007472355	$3.49E^{-12}$	Down
			Unigene8623_All	800	7.66	2.76	1.472676125	0.000034	Down
			Unigene8624_All	2400	2.18	1.38	0.659659868	0.063551591	*
			Unigene8625_All	973	21.59	5.11	2.078968047	$4.49E^{-27}$	Down
			Unigene8626_All	863	2.64	1.09	1.276209795	0.039628154	*
			Unigene8627_All	1008	5.01	1.79	1.484851016	0.000124658	Down
			Unigene12187_All	1708	2.17	1.04	1.061111514	0.013623449	*
			Unigene11772_All	2525	15.02	6.28	1.258048349	$7.69E^{-26}$	Down
			Unigene11773_All	1058	6.07	1.89	1.683310282	0.000000882	Down
			Unigene11774_All	2485	11.96	5.18	1.207193387	$5.85E^{-19}$	Down
C4H	K00487	EC:1.14.13.11	*CL5273.Contig2_All*	1919	22.09	26.13	0.242313706	0.018526896	*
			CL5273.Contig3_All	1960	35.67	30.75	−0.214124805	0.014690933	*
			CL5273.Contig4_All	1945	0.22	0.01	−4.459431619	0.156787001	*
			CL5273.Contig5_All	1923	0.01	0.66	6.044394119	0.0000679	Up
4CL	K01904	EC:6.2.1.12	*CL13908.Contig1_All*	2201	0.44	0.47	0.095157233	0.911900727	*
			CL13908.Contig2_All	2075	10.69	10.5	−0.025872525	0.890011822	*
			CL884.Contig2_All	2043	27.15	24.85	−0.127706346	0.245604132	*
			CL884.Contig3_All	2009	15.66	13.63	−0.20029865	0.165219735	*
			Unigene31064_All	1298	0.94	4.59	2.287761492	$1.88E^{-09}$	Up
CHS	K00660	EC:2.3.1.74	*CL7490.Contig1_All*	1546	60.84	8.05	−2.917959465	$8.07E^{-21}$	Down
			CL7490.Contig2_All	1653	136.44	30.94	−2.140721558	$8.2E^{-14}$	Down
			CL7490.Contig3_All	1519	89.82	6.76	−3.73194157	$1.5E^{-23}$	Down
CHI	K01859	EC:5.5.1.6	*CL11065.Contig2_All*	1000	144.63	21.41	−2.756010135	$2.08E^{-12}$	Down
			CL11065.Contig3_All	1100	79.76	4.92	−4.018935189	$1.15E^{-21}$	Down
F3H	K00475	EC:1.14.11.9	*Unigene19580_All*	1587	66.01	27.43	−1.266929976	$2.43E^{-64}$	Down

F3'H	K05280	EC:1.14.13.21	*CL1781.Contig2_All*	1127	23.67	1.83	–3.693144152	1.41E^{-56}	Down
			CL1781.Contig4_All	1975	13.84	0.91	–3.926833587	3.25E^{-65}	Down
			Unigene21639_All	1946	12.71	5.96	–1.092579795	8.44E^{-13}	Down
F3′5′H		EC:1.14.13.88	*CL3331.Contig1_All*	601	10.46	1.25	–3.064882852	1.53E^{-62}	Down
			CL13432.Contig2_All	1548	8	0.43	–4.217591435	5.05E^{-25}	Down
FLS	K05278	EC:1.14.11.23	*CL1689.Contig1_All*	1376	4.88	2.15	–1.182544488	0.000318551	Down
			CL1689.Contig3_All	1371	5.15	0.79	–2.704647874	5.76E^{-12}	Down
DFR	K13083	EC:1.14.13.88	*CL6896.Contig3_All*	1531	213.84	101.03	–1.08174799	3.12E^{-156}	Down
LDOX	K05277	EC:1.14.11.19	*CL6798.Contig2_All*	1681	143.19	62.5	–1.196002647	6.78E^{-136}	Down
ANR	K08695	EC:1.3.1.77	*CL1364.Contig2_All*	1230	16.63	5.56	–1.580631381	1.88E^{-17}	Down
MYB	K09422		*CL580.Contig2_All*	1203	8.24	3.3	–1.320178313	0.000000976	Down
			CL13134.Contig1_All	1209	0.39	3.78	3.276840205	5.77E^{-11}	Up
			CL13134.Contig2_All	1323	12.26	4.07	–1.590858279	3.35E^{-14}	Down
			Unigene8539_All	1070	5.27	1.84	–1.518097196	0.0000809	Down
MYC	K13422		*CL46956.Contig1_All*	653	15.19	0.82	–4.21135415	4.75E^{-08}	Down
			CL46956.Contig2_All	1921	16.43	0.81	–4.342266762	4.24E^{-80}	Down
			CL46956.Contig3_All	1816	1.81	0.48	–1.914883386	0.000247794	Down

* means that the gene has no difference in red and white coleoptiles.

Table S2. Homologs of *AetMYC1* found in URGI database in *Ae. tauschii* [a].

Database	Accession	Identity	Percentage of identity	E–value	Start	End	Gene	Chromosome
Tauschii v1	TGAC_WGS_tauschii_v1_contig_90597_	668/671	99	0.0	1420	2090	*AetMYC1.4* [21]	2D
Tauschii v1	TGAC_WGS_tauschii_v1_contig_1770231	564/671	84	0.0	1165	529	*AetMYC1.5* [21]	2D
Tauschii v1	TGAC_WGS_tauschii_v1_contig_1766537	258/258	100	$6e^{-129}$	1585	1328	*AetMYC1.4* [21]	2D
Tauschii v1	TGAC_WGS_tauschii_v1_contig_160595	242/258	94	$3e^{-107}$	1142	1399	*AetMYC1.5* [21]	2D
Tauschii v1	TGAC_WGS_tauschii_v1_contig_113746	500/693	72	$2e^{-97}$	11905	12575		
Tauschii v1	TGAC_WGS_tauschii_v1_contig_1031908	493/699	71	$3e^{-83}$	762	1438		
Tauschii v1	TGAC_WGS_tauschii_v1_contig_96755	218/262	83	$2e^{-71}$	1260	1521	*AetMYC2.5* [21]	4D
Tauschii v1	TGAC_WGS_tauschii_v1_contig_108819	462/680	68	$7e^{-59}$	2653	2014	*AetMYC2.5* [21]	4D
Tauschii v1	TGAC_WGS_tauschii_v1_contig_1047480	203/265	77	$1e^{-48}$	266	518		
Tauschii v1	TGAC_WGS_tauschii_v1_contig_98024	144/179	80	$3e^{-38}$	3126	3304	*AetMYC2.6* [21]	4D
Tauschii v1	TGAC_WGS_tauschii_v1_contig_1267503	76/90	84	$1e^{-18}$	226	315		
Tauschii v1	TGAC_WGS_tauschii_v1_contig_1035155	67/75	89	$1e^{-18}$	101	175		
Tauschii v1	TGAC_WGS_tauschii_v1_contig_126603	83/101	82	$1e^{-16}$	188	91		
Tauschii v1	TGAC_WGS_tauschii_v1_contig_1041068	67/78	86	5^{e-16}	411	488		
Tauschii v1	TGAC_WGS_tauschii_v1_contig_1059722	47/51	92	$4e^{-11}$	220	170		

[a] The homologous sequences of *AetMYC1* were searched using BlastN in UIGI (https://urgi.versailles.inra.fr/) database. "Accession" means the contig name. "Identity" indicates number of identical matches/alignment length. "Percentage of identity" means percentage of identical matches. "Start" means the beginning position of matches in the contig, while "End" means the final position of the matches in the contig.

Table S3. The locations of single nucleotide polymorphisms (SNPs) in *AetMYC1* alleles.

Alleles	SNPs		
	786	1309	1434
AetMYC1p	T	G	A
AetMYC1w	C	T	G

AetMYC1r, MG495087; *AetMYC1w*, MG495088.

Table S4. The number of red coleoptile cells after bombardment

Constructs	BCN	RCN	ARCNEC	SD	MaxiRCNEC	MiniRCNEC
pBract214	30	0	0	-	0	0
pBract214:AetMYB7D	30	0	0	-	0	0
pBract214:AetMYC1p	30	0	0	-	0	0
pBract214:AetMYC1w	30	0	0	-	0	0
pBract214:AetMYC1w<br>pBract214:AetMYB7D	30	0	0	-	0	0
pBract214:AetMYC1p<br>pBract214:AetMYB7D	30	1682	56	35	143	10

Notes: BCN, bombardment coleoptile number; RCN, red coleoptile number; ARCNEC, average red cell number per coleoptile; SD, standard deviation; MaxiRCNEC, maximum red cell number per coleoptile; MiniRCNEC, minimum red cell number per coleoptile.

Table S5. Information on *Ae. tauschii* accessions used in this study.

Cultivar	Taxon	Country	Coleoptile color	GenBank accessions	MYB/MYC
CIae1	*Aegilops tauschii*	Pakistan, Baluchistan	red		
CIae2	*Aegilops tauschii*	Pakistan, Baluchistan	red		
CIae3	*Aegilops tauschii*	Afghanistan, Zabul	red		
CIae4	*Aegilops tauschii*	Afghanistan, Ghazni	red		
CIae5	*Aegilops tauschii*	Afghanistan, Baghlan	red		
CIae8	*Aegilops tauschii*	Iran, Mazandaran	red		
CIae9	*Aegilops tauschii*	Iran, Mazandaran	red		
CIae10	*Aegilops tauschii*	Iran, Mazandaran	red		
CIae12	*Aegilops tauschii*	Iran, Mazandaran	red		
CIae17	*Aegilops tauschii*	Iran, Mazandaran	red		
CIae18	*Aegilops tauschii*	Iran, Mazandaran	red		
CIae19	*Aegilops tauschii*	Iran, Mazandaran	red		
CIae20	*Aegilops tauschii*	Iran, Mazandaran	red		
CIae21	*Aegilops tauschii*	Iran, Mazandaran	red		
CIae22	*Aegilops tauschii*	Iran, Gilan	red		
CIae24	*Aegilops tauschii*	Iran, Gilan	red		
CIae25	*Aegilops tauschii*	Iran, Gilan	red		
CIae26	*Aegilops tauschii*	Iran, Gilan	red		
CIae28	*Aegilops tauschii*	Iran, West Azerbaijan	red		
CIae30	*Aegilops tauschii*	Unknown	red		
CIae50	*Aegilops tauschii*	Unknown	red		
CIae51	*Aegilops tauschii*	Unknown	red		
CIae68	*Aegilops tauschii*	Turkey, Kars	red		
CIae71	*Aegilops tauschii*	Unknown	red		

CIae72	*Aegilops tauschii*	Unknown	red
PI210987	*Aegilops tauschii*	Afghanistan, Kondoz	red
PI220326	*Aegilops tauschii*	Afghanistan, Kondoz	red
PI220331	*Aegilops tauschii*	Afghanistan, Faryab	red
PI220642	*Aegilops tauschii*	Afghanistan, Faryab	red
PI317392	*Aegilops tauschii*	Afghanistan, Badghis	red
PI369627	*Aegilops tauschii*	Unknown	red
PI428563	*Aegilops tauschii*	Georgia	red
PI428564	*Aegilops tauschii*	Azerbaijan	red
PI431598	*Aegilops tauschii*	Turkmenistan	red
PI431599	*Aegilops tauschii*	Azerbaijan	red
PI431600	*Aegilops tauschii*	Russian Federation, Dagest	red
PI431601	*Aegilops tauschii*	Azerbaijan	red
PI431602	*Aegilops tauschii*	Turkmenistan	red
PI452131	*Aegilops tauschii*	China, Qinghai	red
PI476874	*Aegilops tauschii*	Afghanistan	red
PI486266	*Aegilops tauschii*	Turkey, Hakkari	red
PI486267	*Aegilops tauschii*	Turkey, Hakkari	red
PI486268	*Aegilops tauschii*	Turkey, Hakkari	red
PI486269	*Aegilops tauschii*	Turkey, Hakkari	red
PI486270	*Aegilops tauschii*	Turkey, Hakkari	red
PI486272	*Aegilops tauschii*	Turkey, Van	red
PI486273	*Aegilops tauschii*	Turkey, Kars	red
PI499262	*Aegilops tauschii*	China, Xinjiang	red
PI499263	*Aegilops tauschii*	Unknown	red
PI499264	*Aegilops tauschii*	France	red
PI499265	*Aegilops tauschii*	Unknown	red
PI508260	*Aegilops tauschii*	China, Xinjiang	red
PI508261	*Aegilops tauschii*	China, Xinjiang	red
PI508262	*Aegilops tauschii*	China, Xinjiang	red
PI508263	*Aegilops tauschii*	China, Shaanxi	red
PI511362	*Aegilops tauschii*	Pakistan, Baluchistan	red
PI511363	*Aegilops tauschii*	Afghanistan, Faryab	red
PI511365	*Aegilops tauschii*	Pakistan, Baluchistan	red
PI511366	*Aegilops tauschii*	Afghanistan, Zabul	red
PI511367	*Aegilops tauschii*	Afghanistan, Kabul	red
PI511368	*Aegilops tauschii*	Iran, Tehran	red
PI511369	*Aegilops tauschii*	Iran, Mazandaran	red
PI511370	*Aegilops tauschii*	Iran, Mazandaran	red
PI511375	*Aegilops tauschii*	Unknown	red
PI511378	*Aegilops tauschii*	Iran, West Azerbaijan	red
PI511379	*Aegilops tauschii*	Iran, West Azerbaijan	red
PI511380	*Aegilops tauschii*	Iran, Mazandaran	red
PI511381	*Aegilops tauschii*	Iran, Mazandaran	red
PI554310	*Aegilops tauschii*	Turkey, Van	red
PI554311	*Aegilops tauschii*	Turkey, Van	red

PI554312	*Aegilops tauschii*	Turkey, Van	red
PI554313	*Aegilops tauschii*	Turkey, Van	red
PI554315	*Aegilops tauschii*	Turkey, Van	red
PI554321	*Aegilops tauschii*	Turkey, Hakkari	red
PI554322	*Aegilops tauschii*	Turkey, Van	red
PI554323	*Aegilops tauschii*	Turkey, Van	red
PI554324	*Aegilops tauschii*	Turkey,Kars	red
PI560532	*Aegilops tauschii*	Turkey, Van	red
PI560533	*Aegilops tauschii*	Turkey, Van	red
PI560534	*Aegilops tauschii*	Turkey, Hakkari	red
PI560536	*Aegilops tauschii*	Turkey, Van	red
PI560538	*Aegilops tauschii*	Turkey,Bitlis	red
PI560755	*Aegilops tauschii*	Turkey, Hakkari	red
PI574466	*Aegilops tauschii*	Georgia	red
PI574467	*Aegilops tauschii*	Rsssian Federation, Dagest	red
PI574469	*Aegilops tauschii*	India	red
PI603221	*Aegilops tauschii*	West Asia	red
PI603223	*Aegilops tauschii*	Iran, Mazandaran	red
PI603224	*Aegilops tauschii*	Russian Federation, Dagest	red
PI603226	*Aegilops tauschii*	Iran, Mazandaran	red
PI603227	*Aegilops tauschii*	Iran, Mazandaran	red
PI603228	*Aegilops tauschii*	Iran, Mazandaran	red
PI603229	*Aegilops tauschii*	Azerbaijan	red
PI603231	*Aegilops tauschii*	Azerbaijan	red
PI603232	*Aegilops tauschii*	Azerbaijan	red
PI603233	*Aegilops tauschii*	Azerbaijan	red
PI603234	*Aegilops tauschii*	Azerbaijan	red
PI603235	*Aegilops tauschii*	Azerbaijan	red
PI603237	*Aegilops tauschii*	Azerbaijan	red
PI603238	*Aegilops tauschii*	Azerbaijan	red
PI603240	*Aegilops tauschii*	Azerbaijan	red
PI603242	*Aegilops tauschii*	Turkmenistan, Balkan	red
PI603244	*Aegilops tauschii*	Iran, Mazandaran	red
PI603245	*Aegilops tauschii*	West Asia	red
PI603246	*Aegilops tauschii*	Portugal	red
PI603249	*Aegilops tauschii*	Iran, Tehran	red
PI603250	*Aegilops tauschii*	Iran	red
PI603251	*Aegilops tauschii*	Iran, Gilan	red
PI603253	*Aegilops tauschii*	Iran, Mazandaran	red
PI603254	*Aegilops tauschii*	Iran, Mazandaran	red
PI603255	*Aegilops tauschii*	Armenia, Erevan	red
PI603256	*Aegilops tauschii*	Azerbaijan	red
PI662053	*Aegilops tauschii*	Turkmenistan	red
PI662055	*Aegilops tauschii*	Turkmenistan	red
PI662058	*Aegilops tauschii*	Turkmenistan	red

PI662060	*Aegilops tauschii*	Turkmenistan	red		
PI662062	*Aegilops tauschii*	Turkmenistan	red		
PI662063	*Aegilops tauschii*	Turkmenistan	red		
PI662064	*Aegilops tauschii*	Turkmenistan	red		
PI662065	*Aegilops tauschii*	Turkmenistan	red		
PI662066	*Aegilops tauschii*	Turkmenistan	red		
PI662067	*Aegilops tauschii*	Turkmenistan	red		
PI662068	*Aegilops tauschii*	Turkmenistan	red		
PI662069	*Aegilops tauschii*	Turkmenistan	red		
PI662095	*Aegilops tauschii*	Tajikistan, Khujand	red		
PI662106	*Aegilops tauschii*	Tajikistan, Khujand	red		
PI662111	*Aegilops tauschii*	Tajikistan, Khujand	red		
PI662112	*Aegilops tauschii*	Tajikistan, Khujand	red		
PI662116	*Aegilops tauschii*	Tajikistan, Khujand	red		
**As60**	***Aegilops tauschii***	**Iran**	**red**	MG495087/MG495089	*AetMYB7D/AetMYC1p*
As61	*Aegilops tauschii*	unknown	red		
As64	*Aegilops tauschii*	unknown	red		
As65	*Aegilops tauschii*	unknown	red		
As71	*Aegilops tauschii*	Xinjiang, China	red		
As74	*Aegilops tauschii*	Shanxi, China	red		
As76	*Aegilops tauschii*	Shanxi, China	red		
**As77**	***Aegilops tauschii***	**Henan, China**	**white**	MG495088/MG495090	*AetMYB7D/AetMYC1w*
As78	*Aegilops tauschii*	unknown	red		
As79	*Aegilops tauschii*	Henan, China	red		
As80	*Aegilops tauschii*	unknown	red		
As82	*Aegilops tauschii*	Henan, China	red		

Table S6. Names and sequences of the primers used in this study.

Number	Primer	Sequence(5'-3')
1	AetMYC1-F	ATGGCGCTGCCAGTAGTTC
2	AetMYC1-R	TCAGCGCCTGCGTATGG
3	AetMYB7D-F	ATGGGGAGGAGGGCGTGCTGTGCCAAGGA
4	AetMYB7D-R	TTAACCGGCCATGTGCAGGGACTTGAGCC
5	Tubulin-F	TGAGGACTGGTGCTTACCGC
6	Tubulin-R	GCACCATCAAACCTCAGGGA
7	AetMYC1attB1-F	AAAAAGCAGGCTTCATGGCGCTGCCAGT
8	AetMYC1attB1-R	AGAAAGCTGGGTTCTCAGCGCCTGCGTAT
9	attB1 adapter	GGGGACAAGTTTGTACAAAAAAGCAGGCT
10	attB2 adapter	GGGGACCACTTTGTACAAGAAAGCTGGGT
